# Genotyping-by-Sequencing Reveals Marker-Based Genome Stability in Tetraploid Clementines for Triploid Breeding

**DOI:** 10.3390/plants15020336

**Published:** 2026-01-22

**Authors:** Pablo Aleza, Andres Garcia-Lor, Pierre Mournet, Luis Navarro, Patrick Ollitrault

**Affiliations:** 1 Centro de Citricultura y Producción Vegetal, Instituto Valenciano de Investigaciones Agrarias (IVIA), Carretera CV-315, km 10.7, Moncada, 46113 Valencia, Spain; garcia_andlor@gva.es (A.G.-L.); lnavarro.ivia@gmail.com (L.N.); 2 AGAP Institute, CIRAD, F-34398 Montpellier, France; pierre.mournet@cirad.fr (P.M.); patrick_ollitrault@hotmail.com (P.O.); 3 AGAP Institute, University of Montpellier, CIRAD, INRAE, Institute Agro, F-34398 Montpellier, France

**Keywords:** citrus, colchicine, adventitious organogenesis, cybrids, SNP markers, allele dosage, haplotype phasing, tissue culture

## Abstract

Tetraploid non-apomictic citrus genotypes are key female parents for 4x × 2x hybridizations aimed at producing seedless triploid hybrids. However, the extent to which different tetraploidization methods affect genome integrity remains insufficiently characterized at a genome-wide scale. In this study, genotyping-by-sequencing (GBS) was used to evaluate marker-based genomic stability in ten tetraploid plants of ‘Clemenules’, ‘Fina’, and ‘Marisol’ clementines obtained via colchicine treatment, in vitro adventitious organogenesis, or somatic cybridization. Diploid parental plants, two haploid plants of ‘Clemenules’ and ‘Fina’ clementines, and one doubled haploid plant of ‘Clemenules’ clementine were included, being the haploid and double haploid essential to resolve allelic phases. After quality filtering, 3333 SNP (Single Nucleotide Polymorphism) markers distributed across the nine citrus chromosomes were identified and used to compare allele dosage patterns along the genome. Across all GBS-covered regions, no major marker-based genomic gains or losses were detected in any tetraploid plant. These results indicate that, at the resolution provided by GBS, all three tetraploidization methods largely preserve chromosome structure, supporting their suitability for citrus triploid breeding programs based on 4x × 2x sexual hybridizations.

## 1. Introduction

Triploid breeding is a key strategy for developing new seedless mandarin cultivars that are demanded by the fresh fruit market, as consumers generally do not accept seedy fruits [1]. Triploid plants are typically considered an evolutionary dead-end, since they usually produce aneuploid gametes with very low fertility [2]. In citrus triploid hybrids, meiosis commonly results in predominantly trivalent associations, together with numerous bivalent and univalent configurations [3]. Additionally, the abortion of megasporogenesis frequently occurs during the period between the first embryo-sac divisions and the fertilized egg cell [4]. For these reasons, citrus triploid hybrids are generally sterile, although they may occasionally produce fruits containing very few seeds.

Triploid citrus plants can be obtained directly from crosses between two diploid genotypes through the union of a 2n megagametophyte with haploid pollen [5,6,7], or from hybridization between diploid and tetraploid parents [8,9,10]. Citrus breeding is hampered by apomixis in most genotypes at both the diploid and tetraploid levels. In citrus, apomixis occurs via adventitious embryony from nucellar cells [11]. Most citrus genotypes are apomictic, with the main exceptions being citrons (*Citrus medica* L.), pummelos (*C. maxima* (Burm.) Merr.), clementines (*C. × aurantium* var. *clementina*) and some mandarin hybrids. In seeds of apomictic citrus genotypes, formation of the nucellar embryos can begin before fertilization [12], and competition between the zygotic and nucellar embryos generally leads to the failure of the zygotic embryo [11,13]. This characteristic represents a major limitation for the use of apomictic tetraploid genotypes as female parents in 4x × 2x hybridizations.

The most effective approach to recover large triploid populations is through 4x × 2x hybridizations using tetraploid non-apomictic genotypes as female parents [10]. This strategy has been little used for triploid breeding due to the difficulty of producing tetraploid plants of non-apomictic genotypes. Clementines are the most important group of mandarins in Spain due to their excellent fruit quality and consumer acceptance. ‘Fina’ is the original clementine cultivar, first described in 1902. It originated as a chance seedling and is now known to result from a hybridization between ‘Willow leaf’ mandarin (*C. reticulata* var. *deliciosa*) and sweet orange (*C. x aurantium* var. *sinensis*) [14]. Spontaneous mutations of ‘Fina’ clementine, along with additional and subsequent mutations accumulated on the derived clementines, have originated all known clementine varieties. ‘Clemenules’ was obtained in 1953 in Nules (Castellón, Spain) from a spontaneous mutation of ‘Fina’, which is one of the most important clementine varieties in the Spanish citrus industry due to its fruit quality and remains the most widely cultivated clementine in Spain. ‘Marisol’ originated in 1970 in Bechí (Castellón) from a spontaneous mutation of ‘Oroval’, which itself arose as a spontaneous mutation of ‘Fina’. The production of tetraploid plants of clementines is of great interest for triploid breeding because they produce fruits of excellent quality and are non-apomictic genotypes, allowing the production of large progenies of triploid hybrids in a relatively straightforward manner.

Spontaneous chromosome doubling in nucellar tissue is the primary mechanism underlying tetraploidization in apomictic citrus genotypes [15]. However, this phenomenon does not occur in non-apomictic genotypes, making it necessary to employ alternative methodologies to recover tetraploid plants from these genotypes. Aleza et al. [16] developed an efficient method for producing stable tetraploid plants of non-apomictic citrus genotypes by combining in vitro shoot-tip grafting with colchicine and/or oryzalin treatment of shoot-tips. When applied to adult plant material, this technique avoids the long juvenile phase associated with tetraploid recovery from nucellar seedlings. The recovery of citrus cybrid plants as a by-product of symmetric protoplast fusion has also been reported, yielding mainly diploid cybrids [17,18,19] and much less frequently, tetraploid cybrids [20,21]. Tetraploid cybrids arise from protoplast fusion between one protoplast from the callus parent and two diploid protoplasts from the leaf parent, followed by failed nuclear fusion, the subsequent loss of the callus-parent nucleus and the incorporation of mitochondria released from ruptured embryogenic cells into the fused leaf protoplasts [20]. The use of non-apomictic citrus genotypes as leaf parents enables the recovery of tetraploid cybrids that produce non-apomictic seeds, although these tetraploid cybrids display juvenile characteristics as they originate from embryogenesis [21].

Somaclonal variation is a useful approach for citrus improvement and has been applied for this purpose in several laboratories [22,23,24]. Clementines are good candidate for somaclonal variation studies because they are genetically unstable and frequently produce budsports in the field. We attempted to recover somaclonal variants through in vitro adventitious organogenesis from adult internodal segments and plant regeneration via shoot-tip grafting. Following this methodology, we regenerated more than 450 plants all which were diploid except for two tetraploid plants of ‘Clemenules’ clementine (unpublished data).

Somatic hybridization by protoplast fusion has been reported to induce genomic instability in citrus and related species [19], while in vitro adventitious organogenesis has been shown to promote somaclonal variation in sweet orange [24]. Somaclonal variation constitutes an important source of heritable diversity, encompassing both genetic mutations and epigenetic modifications, including alterations in DNA methylation patterns and transposable element activity [25,26]. In addition, colchicine treatments have been reported to induce genetic and chromosomal variations in several plant species, including cotton (*Gossypium* spp.) [27], *Brachiaria* spp. [28,29], *Lilium* spp. [30], and *Calendula officinalis* [31]. Based on these observations, we aimed to determine whether these techniques could similarly induce genetic instability in tetraploid citrus plants. Genetic analyses of citrus tetraploids plants have traditionally relied on a limited number of molecular markers [16,24,25,26,27], providing only partial genome coverage and limiting the detection of structural chromosomal variations, such as large deletions or duplications affecting heterozygosity. Genotyping-by-sequencing (GBS) has been successfully applied to investigate the phylogenomic structure of triploid and tetraploid citrus genotypes [32,33] and to identify chromosomal instabilities in allotetraploid somatic hybrids obtained by protoplast fusion [19]. However, as highlighted by Ahmed et al. [32], GBS alone does not allow accurate estimation of allele dosage at individual SNP loci in polyploid genomes. To overcome this limitation, these authors proposed an approach based on read-depth information for reference and alternative alleles across successive phased SNPs. In this context, the availability of genotyping data from haploid and doubled haploid plants provides comprehensive phasing information across the clementine genome. The development of haploid and doubled haploid plants therefore represents a valuable resource for genetic and genomic studies, and significant efforts have been made to generate such materials in various tree species [34,35].

In the present study, we expanded beyond conventional marker approaches by performing a genotyping by sequencing (GBS) analysis of tetraploid clementine plants recovered through the three methods described above (in vitro shoot-tip grafting and colchicine treatment, in vitro adventitious organogenesis from adult internodal segments and plant regeneration via shoot-tip grafting, and somatic cybridization) together with their diploid parental genotypes. Our main objective was to determine whether any of these methodologies induced gains or losses of large chromosome regions.

## 2. Results and Discussion

### 2.1. SNP Mining

After genotype calling against the Valencia sweet orange DVS_B genome reference and filtering for diallelic SNPs with a minimum allele frequency of 0.01, we identified a total of 15,480 SNPs. Further filtering for markers without missing data, heterozygous in the three diploid clementines and with the same homozygous allelic configuration as the haploid and doubled-haploid ‘Clemenules’ clementine, resulted in the selection of 3333 SNPs (Table S1).

### 2.2. Assessment of Allele Dosage

To identify potential variations along the genome, the first step of the analysis was to study the allele read frequencies for SNPs across the genome. For the haploid and doubled-haploid plants, the theoretical frequencies are 0 and 1, whereas for diploid and tetraploid plants the expected frequency is 0.5 in the absence of large structural variation, because only heterozygous markers were selected for the clementines.

**Figure 1:**
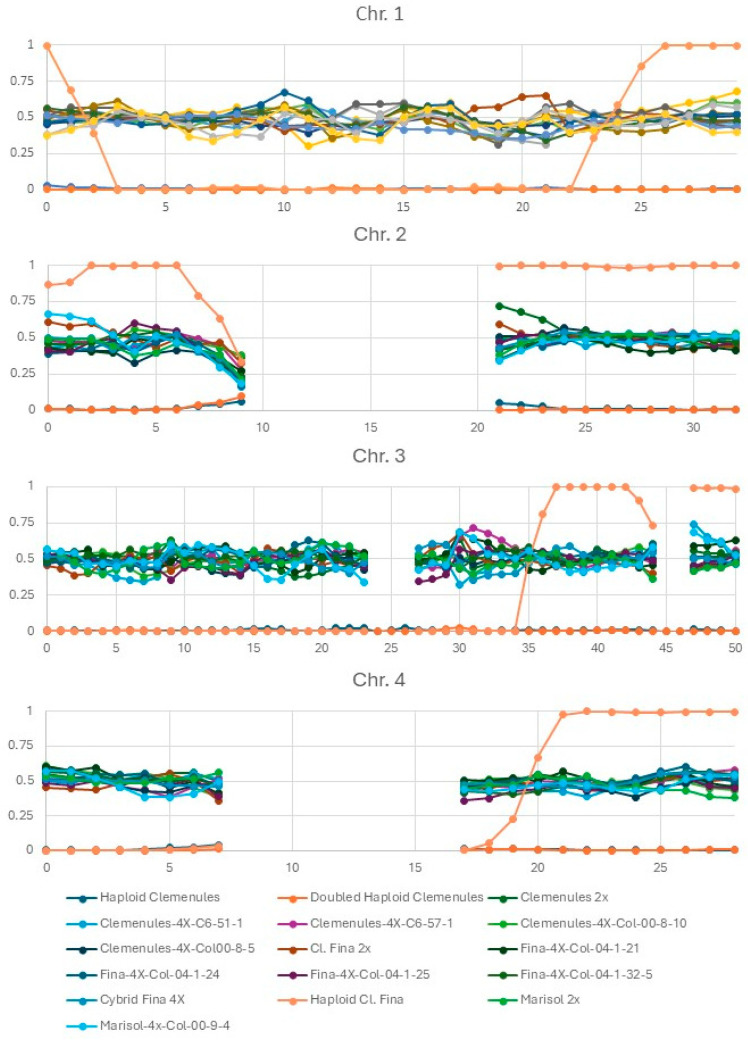
Allele frequencies observed along chromosomes 1 to 4 in all analyzed haploid, doubled haploid, diploid, and tetraploid plants. The *y*-axis represents the frequency of the phased alternative allele. A frequency of 0.5 corresponds to the three diploid parental clementine genotypes and the ten tetraploid plants, whereas frequencies ranging from 0 to 1 correspond to haploid and doubled haploid plants of the ‘Fina’ and ‘Clemenules’ clementines. The *x*-axis represents chromosome length, shown in 1 Mb intervals.

**Figure 2:**
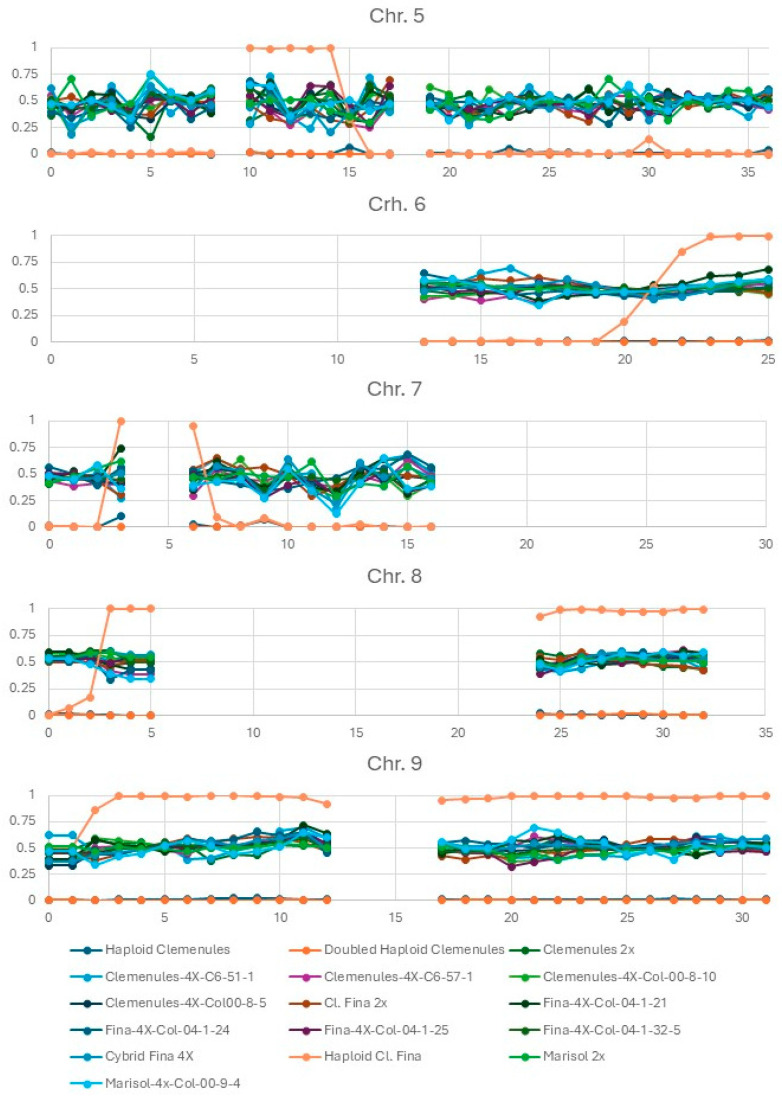
Allele frequencies observed along chromosomes 5 to 9 in all analyzed haploid, doubled haploid, diploid, and tetraploid plants. The *y*-axis represents the frequency of the phased alternative allele. A frequency of 0.5 corresponds to the three diploid parental clementine genotypes and the ten tetraploid plants, whereas frequencies ranging from 0 to 1 correspond to haploid and doubled haploid plants of the ‘Fina’ and ‘Clemenules’ clementines. The *x*-axis represents chromosome length, shown in 1 Mb intervals.

The analysis was conducted using the 3333 selected SNPs that were heterozygous in all three parental clementine varieties. After phasing these SNPs according to the haploid/doubled-haploid ‘Clemenules’, the frequency of the clementine haplotype alternative to the one of the haploid/doubled-haploid ‘Clemenules’ was estimated using successive windows of 1 Mb. When fewer than 10 SNPs were present in a 1 Mb segment, the segment was considered uninformative (missing data). Large gaps lacking information were observed on chromosomes 2, 4, 6, 7, 8 and 9 (Figure 1 and Figure 2). Most of these regions with few or no markers in our analysis correspond to clementine genomic regions identified by [14] as fully homozygous due to inbreeding. Consequently, only heterozygous regions provided informative data regarding parental haplotype dosage and, by extension, potential variation in local ploidy. Within these informative regions, we did not detect genomic regions exhibiting deviations in tetraploid plants compared to the range of allelic ratio variability observed in the diploid parental plants, nor any loss or gain of chromosomal fragments in tetraploid plants recovered using any of the three methodologies (Figure 1 and Figure 2). However, our GBS method, based on Apek1 digestion, did not provide information on the entire genome and focused on genic regions of the genome. Therefore, we cannot exclude the possibility of variations in repetitive regions, particularly centromeric regions, which are sparsely covered by our markers. In addition, point mutations such as SNPs or small indels were not examined.

Finally, it is important to note that the doubled-haploid plant of ‘Clemenules’ clementine shows identical allele frequencies across all SNP markers and chromosomes, consistent with its origin from a budsport spontaneous chromosome doubling. The GBS analysis also reveals two clearly distinct genomic profiles between the haploid plants of ‘Clemenules’ and ‘Fina’, revealing the different crossover breakpoints that occurred during the formation of the two distinct gametes that gave rise to each haploid plant. (Figure 1 and Figure 2).

### 2.3. Genomic Stability of Recovered Tetraploid Plants

Colchicine treatment is one of the most widely used methods for inducing polyploidy in plants, as it disrupts spindle fiber formation during cell division, leading to chromosome doubling [36]. In citrus, [16,37,38] tetraploid plants have been obtained using this approach. Aleza et al. [16] obtained, by shoot-tip grafting and subsequent colchicine treatment, four tetraploid plants of ‘Fina’ clementine (Col-04-1-21, Col-04-1-24, Col-04-1-25 and Col-04-1-32-5), two tetraploid plants of ‘Clemenules’ clementine (Col-00-8-05 and Col-00-8-10) and one tetraploid plant of ‘Marisol’ clementine (Col-00-9-4), that have been used in this study. Recently, other methods [15] have been developed based on in vivo colchicine application to the decapitated epicotyl juvenile seedlings, enabling tetraploid induction and the recovery of stable tetraploid plants of non-apomictic mandarins, pummelo and citron genotypes. Nevertheless, this approach results in tetraploid hybrid plants that retain juvenile characteristics, requiring several years before they can be used as parental material in new genetic combinations. Conversely, applying shoot-tip grafting and colchicine treatment to mature tissues markedly shortens the time needed before these plants can be utilized as parents in subsequent breeding programs.

Colchicine treatment has been shown to induce chromosome loss or rearrangements, as well as gene mutations, in different crop species such as flax, sunflower, barley and cotton [27,39,40]. It has also been reported that the treatment of explants with colchicine may cause some chromosomal aberrations, leading to the formation of aneuploid population in grapes [41]. In *Lilium davidii* var. *unicolor*, Li et al. [30] analyzed diploid and colchicine-induced tetraploid plants with five ISSR (Inter-simple sequence repeat) markers and identified polymorphisms between ploidy levels for all markers analyzed. El-Nashar and Ammar [31] also detected genetic variability among calendula plants exposed to different colchicine concentrations using SRAP (Sequence-Related Amplified Polymorphism) markers, which are used to amplify coding regions of DNA with primers targeting ORFs. In the present study, we did not identify any loss or gain of chromosomal fragments in tetraploid plants recovered through colchicine treatment.

Somatic hybridization through callus and leaf protoplast fusion is a technique used in citrus to generate allotetraploid hybrids and tetraploid cybrids from both apomictic and non-apomictic genotypes [19,20,21,42,43]. Citrus cybrids have been obtained from more than 40 combinations [19,21,42], but there are few references on regeneration of tetraploid cybrids. Grosser et al. [43] regenerated 99 plants following polyethylene glycol-mediated protoplast fusion between embryogenic callus of ‘Nova’ tangelo (*C. x aurantium* var. *clementina* X (*C. x aurantium* var. *tangerina ined*. X *C. x aurantium* var. *paradisi*)) and leaf protoplasts of ‘Succari’ sweet orange (*C. x aurantium* var. *sinensis*). Of these regenerants, only one plant was identified as a tetraploid cybrid of ‘Succari’ sweet orange. Subsequently, Guo et al. [20] recovered more than 150 plants via electrofusion between embryogenic callus of ‘Page’ tangelo (*C. x aurantium* var. *clementina* X (*C. x aurantium* var. *paradisi* x *C. x aurantium* var. *tangerina ined*.) and leaf protoplasts of rough lemon (*C. x limonia* var. *jambhiri*), yet only a single tetraploid cybrid was obtained. Similarly, Aleza et al. [21] produced 26 plants through electric protoplast fusion between embryogenic callus of ‘Chios’ mandarin and leaf protoplasts of Clementine, of which only one was confirmed as a tetraploid cybrid. These studies collectively demonstrate that tetraploid cybrid formation in citrus occurs at a very low frequency. Both diploid and tetraploid cybrids possess the nuclear genome of the leaf parent and the mitochondrial genome from the embryogenic callus parent, while the chloroplast genome is inherited randomly.

The characterization of regenerated allotetraploid and somatic cybrid plants relies on multiple complementary approaches, including morphological evaluation, cytological determination of ploidy level (via chromosome counting and flow cytometry), and molecular analysis using diverse DNA markers [21,42]. Individually, these techniques are insufficient to elucidate the somatic cybrid origin; therefore, an integrated methodological framework is required. In particular, SSR markers have proven especially valuable for characterizing the nuclear and organellar genomes of somatic cybrids. In a previous study, we recovered 28 cybrid plants derived from the embryogenic callus parent ‘Chios’ mandarin and the leaf parent Clementine through electrofusion of protoplasts. Flow cytometry revealed that all regenerated plants were diploid except for a single tetraploid plant. This tetraploid cybrid was further characterized using twenty nuclear SSR markers, three mitochondrial markers, and two chloroplast markers [21]. The analyses revealed that the nuclear genome corresponded to that of the Clementine leaf parent, and no polymorphisms were detected in either the mitochondrial or chloroplast genomes.

In the present study, we further analyzed this tetraploid cybrid using GBS to identify potential gains or losses of genomic regions; however, no such variations were detected. It is noteworthy that tetraploid cybrids and allotetraploid somatic hybrids exhibit juvenile characteristics because they are regenerated through embryogenesis, whereas tetraploids derived from colchicine-treated adult tissues or regenerated through in vitro organogenesis do not display this limitation. The latter are therefore of greater interest from a breeding standpoint, as they can be used immediately as parental material to produce new genetic combinations, thereby increasing the overall efficiency of our breeding program.

The chromosomal stability observed in our study is consistent with the identical nuclear genomes reported by Dambier et al. [19] for the five cybrids they obtained, which were indistinguishable from their respective leaf parents. Moreover, in allotetraploid somatic citrus hybrids recovered through protoplast fusion, different authors identified frequent chromosomal instability and concluded that an important part of these variations in allotetraploid plants may occur during the induction and multiplication of embryogenic calluses used for protoplast isolation or variation promoted during plant regeneration from isolated protoplasts via somatic embryogenesis [19]. Such genomic changes have also been observed in interspecific somatic hybrids of different *Brassica* species [44], *Nicotiana tabacum* L. + *Atropa belladonna* L. somatic hybrids [45], in symmetric somatic hybrids between *Arabidopsis thaliana* L. and *Bupleurum scorzonerifolium* Willd [46], somatic hybrids between bread wheat and other grass species [47], and in somatic hybrid cells between *Oryza sativa* L. subsp. *japonica* + *O. sativa* L. subsp. *indica* and between *Japonica* rice and bread wheat (*Triticum aestivum* L.) [48].

In vitro adventitious organogenesis from internodal segments of adult material is a methodology used by only a few research groups for the genetic transformation of citrus plants [49]. In our laboratory, this approach has been applied to induce somaclonal variation in different clementine genotypes. More than 450 plants were regenerated, all of which were diploid except for two tetraploid plants of ‘Clemenules’ clementine. The diploid ones were cultivated in experimental plots at IVIA (Moncada, Valencia, Spain), where no significant differences were observed in either the ripening period or fruit size, as these traits are easily assessed in the field when compared with their parental counterparts. The two tetraploid plants may have arisen either during the in vitro adventitious organogenesis process or from internodal tissues that underwent a somatic mutation leading to chromosome doubling, as previously reported for the doubled haploid plant of ‘Clemenules’ clementine. Chromosome doubling in citrus somatic tissues was observed by Raghuvanshi [50]; however, very few tetraploid budsports have been identified due to unfavorable competition between diploid and tetraploid cells in the meristem [51]. Our GBS analysis does not allow us to confirm either of the previously proposed hypotheses regarding their origin. Nonetheless, regardless of the biological process responsible for their origin, we can confirm that neither of the two tetraploid plants obtained through in vitro adventitious organogenesis exhibits any gain or loss of chromosomal fragments.

## 3. Materials and Methods

**Plant material**

A total of ten tetraploid plants were analyzed in this study, along with their respective diploid clementine genotypes, ‘Clemenules’ (IVIA-022), ‘Fina’ (IVIA-039) and ‘Marisol’ (IVIA-093) grown at IVIA Citrus Germplasm Bank located in Moncada, Valencia, Spain. In addition, we analyzed the haploid plant of ‘Clemenules’ clementine (IVIA-638) that was selected by the International Citrus Genomic Consortium (ICGC) to establish the reference sequence of the nuclear genome of citrus [14], and a doubled haploid plant of this genotype derived from spontaneous chromosome doubling. We also included a haploid plant of ‘Fina’ clementine obtained previously in our laboratory.

The tetraploid plants used in this study included four tetraploid plants of ‘Fina’ clementine (Col-04-1-21, Col-04-1-24, Col-04-1-25 and Col-04-1-32-5), two tetraploid plants of ‘Clemenules’ clementine (Col-00-8-05 and Col-00-8-10) and one tetraploid plant of ‘Marisol’ clementine (Col-00-9-4), all obtained via shoot-tip grafting followed by colchicine treatment, according to the methodology described by Aleza et al. [16]. Two additional tetraploid plants of ‘Clemenules’ clementine (C6-51-1 and C6-57-1), generated through in vitro adventitious organogenesis from internodal segments, were also included, as well as one tetraploid cybrid of ‘Fina’ clementine (Cybrid Fina) obtained by protoplast fusion [21].

**Plant Genotyping**

Genomic DNA was isolated using the Plant DNAeasy^®^ kit (Qiagen, Hilden, Germany), following the manufacturer’s instructions. The DNA concentration was adjusted to 20 ng/µL, and the ApekI (New England Biolabs, Hitchin, UK) GBS libraries were prepared according to the protocol described by Elshire et al. [52]. Digestion was performed at 75 °C for 2 h, followed by enzyme inactivation at 65 °C for 20 min. The ligation reaction was carried out in the same plate as the digestion, using T4 DNA ligase (New England Biolabs, Hitchin, UK) at 22 °C for 1 h; the ligase was inactivated prior to pooling the samples by holding it at 65 °C for 20 min. For each library, ligated samples were pooled and PCR-amplified in a single tube. Complexity was further reduced using PCR primers with one selective base (A), as described by Sonah et al. [53]. Pair-end sequencing was performed on a single lane of an Illumina HiSeq4000 at Genewiz facilities (Leipzig, Germany). Raw sequencing data were demultiplexed with GBSX v1.3 [54]. Cutadapt v1.8 [55] was used to remove the adapter sequences and low-quality bases (-q 20,20). Processed reads shorter than 30 were discarded. SNP genotype calling was then performed with the VCF-Hunter 2.1.0 pipeline (https://github.com/SouthGreenPlatform/VcfHunter accessed on 11 November 2025) as described in Baurens et al. [56], using VcfPreFilter.1.0.py and vcfFilter.1.0.py utilities and the genome assembly of Valencia sweet orange DVS_B genome v1.0 [57] as reference. For genotype calling, positions with less than five reads were considered as missing data. Polymorphic sites were then filtered for diallelic SNPs with minor allele frequencies (MAFs) greater than 0.01.

**Allelic dosage analysis**

For polyploids, GBS does not allow for accurate estimation of allele dosages at a single SNP locus [32]. Ahmed et al. [32] developed an efficient approach based on the frequency of reads of phased successive markers to estimate the haplotypic doses in successive genomic windows of at least 10 markers. The published tool, called TraceAncestor, was successfully applied to analyze the phylogenomic structure of *Citrus* polyploid germplasm [32], to perform genetic studies of tetraploid progenies resulting from interspecific 4x × 4x crosses and to identify chromosome instability in tetraploid somatic hybrids [19].

The present analysis was based on a total of 3333 SNP markers heterozygous in the three parental diploid clementines, considering genomic windows of 1 Mb as locus for allele dose estimation. Frequencies of the reference and alternative alleles were calculated by dividing the number of reads each within each 1 Mb region by the total number of reads. For this analysis, we leveraged the genotyping data generated for the haploid and doubled haploid clementines, providing the phase information across the clementine genome, except in the vicinity of the eight recombination breakpoints identified for the clementine gamete that produced the sequenced haploid plant [58]. Additionally, this approach enabled the identification of differences in breakpoint positions between the gametes that generated the ‘Fina’ and ‘Clemenules’ haploid plants.

## 4. Conclusions

GBS alone does not allow precise estimation of allele dosage at individual SNP *loci* in polyploid genomes. To address this limitation, 3333 SNPs heterozygous in all three parental clementine varieties were phased based on the haploid and doubled-haploid ‘Clemenules’. The frequency of the clementine haplotype alternative to that of the haploid/doubled haploid ‘Clemenules’ clementine was then calculated using successive windows of 1 Mb across the genome. Using this approach, no evidence of chromosomal instability was detected during clementine tetraploidization, regardless of the tetraploidization method employed. This result indicates that GBS is a useful methodology to analyze the genetic stability of tetraploid plants produced by colchicine treatment, in vitro adventitious organogenesis, and somatic cybridization, providing more detailed genome-wide information than that obtained with traditional molecular markers such as SSRs, ISSRs or SRAPs.

These tetraploid plants of ‘Clemenules’, ‘Fina’ and ‘Marisol’ clementines serve as valuable female parents for producing large progenies of new triploid hybrids. Indeed, these tetraploid clementine plants, artificially generated in the laboratory, have facilitated the selection of 19 triploid mandarin hybrids with excellent organoleptic properties. Notably, one of these hybrids, ‘Vera’ mandarin, produced by sexual hybridization between tetraploid ‘Clemenules’ clementine as the female parent and ‘Nova’ tangelo as the male parent, is expected to be released to the Spanish citrus industry in the near future.

## Data Availability

The original contributions presented in this study are included in the article/Supplementary Material. Further inquiries can be directed to the corresponding author.

## Supplementary Materials

The following supporting information can be downloaded at: https://www.mdpi.com/article/10.3390/plants15020336/s1, Table S1: 3333 SNPs markers heterozygous for clementines used to analyze allele dosage and chromosome stability of tetraploid clementines. Frequencies of the reference and alternative alleles were calculated dividing the number of reads for reference allele and alternative allele.
